# A wet dressing for male genital surgery: A phase II clinical trial

**DOI:** 10.1590/S1677-5538.IBJU.2016.0109

**Published:** 2016

**Authors:** Fábio de Oliveira Vilar, Flávia Cristina Morone Pinto, Amanda Vasconcelos Albuquerque, Ana Gabriela Santos Martins, Luiz Alberto Pereira de Araújo, José Lamartine de Andrade Aguiar, Salvador Vilar Correia Lima

**Affiliations:** 1 Serviço de Urologia do Hospital das Clínicas, Departamento de Cirurgia do Centro de Ciências da Saúde da Universidade Federal de Pernambuco, UFPE, Brasil; 2 Departamento de Cirurgia do Centro de Ciências da Saúde da Universidade Federal de Pernambuco, UFPE, Brasil; 3 Serviço de Cirurgia Pediátrica do Hospital das Clínicas, Departamento de Cirurgia do Centro de Ciências da Saúde da Universidade Federal de Pernambuco, UFPE, Brasil

**Keywords:** Safety, Surgical Procedures, Operative, Genitalia, Male

## Abstract

**Purpose:**

This study was to confirm the safety and efficacy of BC dressing when used in surgical male wound healing at the urogenital area.

**Methods:**

Open, non-controlled clinical study of phase II. A total of 141 patients, among those children, adolescents and adults with hypospadias (112), epispadias (04), phymosis (13) and Peyronie's disease (12) that had a BC dressing applied over the operated area after surgery. A written informed consent was obtained from all participants. Study exclusion criteria were patients with other alternative treatment indications due to the severity, extent of the injury or the underlying disease. The outcomes evaluated were efficacy, safe and complete healing. The costs were discussed.

**Results:**

In 68% patients, the BC dressing fell off spontaneously. The BC was removed without complications in 13% of patients at the outpatient clinic during the follow-up visit and 17% not reported the time of removal. In 3% of the cases, the dressing fell off early. Complete healing was observed between 8th and 10th days after surgery. The BC dressings have shown a good tolerance by all the patients and there were no reports of serious adverse events.

**Conclusion:**

The bacterial cellulose dressings have shown efficacy, safety and that can be considered as a satisfactory alternative for postoperative wound healing in urogenital area and with low cost.

## INTRODUCTION

Surgical correction of genitalia anomalies has evolved recently with the use of new techniques, instruments and sutures, which have contributed to better results. In spite of this, the correct choice of dressing is still challenging, because wound healing is a dynamic and complex phenomenon and its phase duration in the urogenital area is longer when compared to dermatological healing. A number of materials have been used with different results, but there is no consensus about their use (1–7). Thus, the question remains regarding which dressing is the best for postoperative male wound healing.

The clinical indication of a specific dressing is based upon the protective function and mechanical barrier of the tissues against contamination and reduction of edema caused by the surgical trauma. The material to be used should have physical features including elasticity, resistance and flexibility and must adjust tightly to the tissue surface. The chosen dressing must present minimal adverse reactions when in contact with living tissues or organic fluids and must be easily removable (8, 9).

A bacterial cellulose (BC) dressing is being studied as a viable alternative. Previous studies, phase I, have shown its effectiveness as a mechanical barrier and a safe adjuvant in the treatment of a surgical wound after hypospadias correction. This material showed the following characteristics for an ideal dressing: it removed exudates, created a moist environment, offered protection from foreign substances and promoted tissue regeneration (9).

The objective of this study was to confirm the safety and efficacy of the BC dressing when used in surgical male wound healing in the urogenital area.

## MATERIALS AND METHODS

### Sample

This was an open, non-controlled clinical trial (Non-Randomized Controlled Trial, NRCT), phase II study, to assess the BC dressing efficacy and safety. Both efficiency and safety were demonstrated in a preview study, phase I (Randomized Controlled Trial, RCT). The phase I study, using polyurethane and BC dressings enabled comparison and exclusion of the polyurethane group, due to better results obtained from the BC dressing in cases of hypospadias repair (9).

The phase ll population study included children, adolescents and adults with hypospadias (112–40% proximal and 60% distal), epispadias [04], phymosis [13] and Peyronie's disease [12]. The patients and their relatives were formally informed about the study and were invited to join it. One hundred and forty-one [141] patients were included in the study after a written informed consent.

Patients were submitted to anamnesis, including questions about previous surgeries, and physical and urological examinations. The exclusion criteria were patients with other alternative treatment indications due to the severity, extent of the injury or due to underlying disease.

### Technique of BC dressing application

A physician, with residents participating as assistants, operated on the patients. Immediately after the end of the surgery the BC dressing was applied. To make sure that all procedures were carried out properly, all patients were hospitalized in a public teaching hospital for 10 days for hypospadias or epispadias and 2 days for Peyronie disease. Circumcision patients left the hospital on the same day. The BC dressing, sized 8.0×15.0cm, could be cut according to patient surgical wound size. The BC dressings had been previously sterilized using 25kGy of gamma irradiation. After end of the surgical procedure, the area was washed out with saline and dried with gauze. The dressings were applied over the whole penile shaft excluding glands in all cases. No tension was applied to the wound area when the BC dressing was fixed (9). The BC dressings were donated by the Laboratory of Biopolymers at the Experimental Station of Sugarcane, Federal Rural University of Pernambuco.

### Outcomes evaluated

Safety was assessed by adverse event reports, such as skin irritability next to the dressing area, categorized by feeling of warmth, itching, swelling, pain, and hyperemia.

Efficacy was classified according to the degree of adhesion to the wound area (fully adhered, partially detached or without adhesiveness); discomfort (described during the questionnaire as “very uncomfortable”, “uncomfortable”, or “not a problem” during the BC dressing use); and transudation, evaluated by exudates drainage and the BC dressing ability to remain fixed when wet.

The primary endpoint was the time range needed for complete healing, measured by time at which the BC dressing spontaneously fell off. Other important outcomes such as pain, wound volume reduction, granulation, odor and scar quality were also monitored.

In addition to these parameters, a comparison with other dressings was made in order to estimated costs and relationship of BC to other dressing already available on the market. The comparisons ranged from individual sale price for each dressing to the required number of surgical wound manipulations required in the urogenital area, over the 14 days that the patient was being monitored.

Furthermore, the study also provides a descriptive analysis of phase II data. It compares statistical inference between phases I and II using the Fisher's exact test, considering the nominal level of 0.05 to reject the null hypothesis.

### Ethical Aspects

The study followed the ethical recommendations of the National Council of Health, the Helsinki Declaration and the Nuremberg Code for human experiment. The study is also listed on www. clinicaltrials.gov, #NCT02531828, and was approved by the National Ethics Committee in Research (CONEP #676.414). The non-inclusion of a control group was discussed and accepted by the Ethical Committee of the Institution.

## RESULTS

Table-1 summarizes the results. The groups ranged in ages from 2 to 12 years old (children), with an average of 5.4 years (hypospadias or epispadias); 12 to 16 years old (adolescents) with an average of 14.7 years (circumcision); and 50 to 60 years old (adults), with an average of 53.6 years (Peyronie's disease).

**Table 1:** Sample characterization and parameters evaluated.

Disease	N	Hospitalization (N days)	Age (average/years)	Time to Dressing Removal (day)				
				Fell off Spontaneously			Removed	Not Informed
				1st*	9th - 10th	11th - 14th	8th – 10th	
Hypospadias	112	10	5.4	2	66	25	0	19
Epispadias	4			0	0	3	0	1
Phymosis	13	0	14.7	2	0	1	6	4
Peyronie's	12	2	53.6	0	0	0	12	0
**Total**	**141**	**10**		**4**	**66**	**29**	**18**	**24**
(%)				**3%**	**47%**	**21%**	**13%**	**17%**

**Note: N** = number. Values are average, total number or percentage (%).

* The dressing was reapplied and remained until 11th day.

The follow-up evaluation was done by one of the team members on a daily basis during the time the patients were in hospital. The BC dressing removal was carried out considering the monitoring time previously defined for each case.

The patients and their parents were advised to keep the bandage in situ after hospital discharge and to inform the researchers when the BC dressing fell off spontaneously.

**Figure 1:**
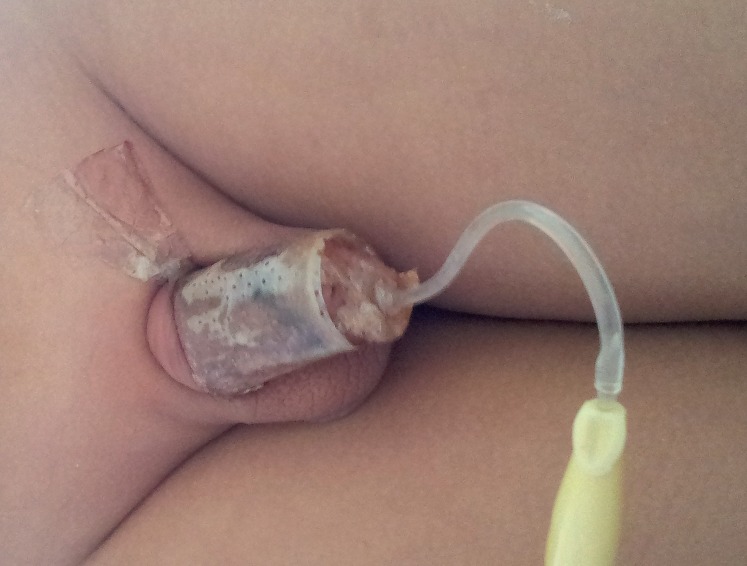
BC dressing molded to the penile shaft, involving all surface.

In 66 cases, the dressing fell off spontaneously around the 9^th^ or 10^th^ day during the hospital stay. These patients were part of the hypospadias correction group (Figure-1).

**Figure 2:**
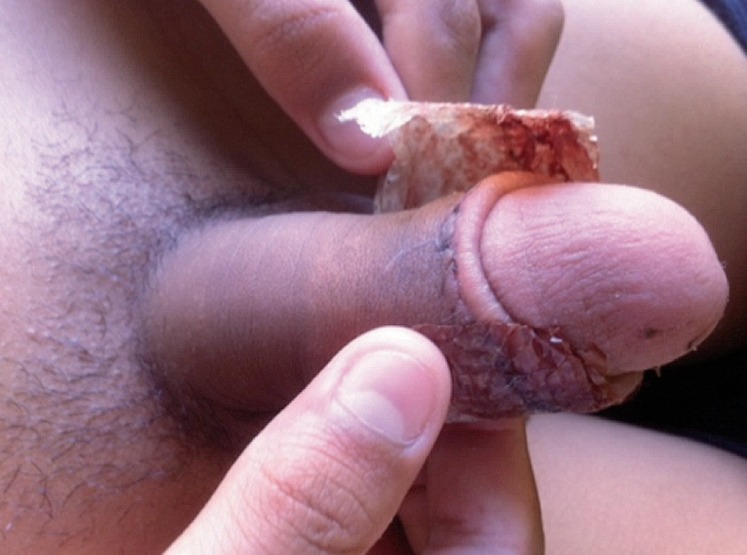
BC dressing fell off spontaneously, after complete healing.

For twenty-nine patients the BC dressing fell off between 11 to 14 days post surgery, with the regular washing instructions having been followed properly. These patients underwent surgery for hypospadias correction, epispadias or phimosis (Figure-2).

Six adult patients submitted to circumcision and all patients treated for Peyronie's disease [12] had their BC dressing removed at the clinic during the follow-up visit, between the 8th and 10th days after surgery. In all cases [18] the operated area was perfectly healed.

The exact time of spontaneous falling of the dressing was not reported for twenty four (17%) patients, but all of them were evaluated during the follow-up visit and a satisfactory outcome was observed.

In four patients (2 phymosis, 2 hypospadias) the BC dressing fell off spontaneously on the first postoperative day without further complications except edema. In those cases, the dressing was reapplied and remained until the 11^th^ day. The BC dressings were well tolerated by all patients. BC efficacy and safety in all cases are summarized in Table-2. Hyperemia limited to the dressing area, but without edema or other local irritation signs was observed in five patients.

**Table 2:** Outcomes evaluated in Phase II compared to Phase I study (unpublished data).

Outcomes		Phase I Study (Hypospadias)		Phase II Study (Hypospadias, Epispadias, Phymosis and Peyronie's Disease)			
		Polyurethane	BC	BC	p-value	RR	IC 95%
	N° of patients	30	30	141	-	-	-
SAFETY	Adverse events:						
	Hyperemia	0	1 (3.3%)	5 (3.6%)	0.6120^a^	1.556	0.8304–2.914
	Hyperemia/Edema	2 (6.7%)	0	0	0.1471^b^	2.264	0.8678–5.906
	Edema	1 (3.3%)	0	0	1.0000^c^	0.9483	0.1537–5.852
EFFICACY	Adherence degree:						
	Fully adhered	30 (100%)	28 (93.3%)	137 (97.2%)	-	-	-
	Partially detached	0	2 (6.7%)	2 (1.4%)	0.4915^a^	-	-
					1.0000^b^	-	-
					0.1453^c^	0.3394	0.1204–0.9571
	Difficulty removed	3 (10%)	0	0	0.2373^a^	2.111	1.606–2.776
					0.0050^b^ *	6.222	4.404–8.791
	Early dressing fall	0	2 (6.7%)	4 (2.8%)	0.4915^a^	-	-
					1.0000^b^	-	-
					0.3105^c^	0.5419	0.1664–1.765
	Pain and discomfort:						
	Uncomfortable	23 (76.7%)	24 (80%)	88 (62.4%)	1.0000^a^	0.9088	0.5078–1.626
	Not a problem	7 (23.3%)	6 (20%)	53 (37.6%)	0.2054^b^	1.776	0.8095–3.897
					0.0898^c^	2.107	0.9120–4.868
	Use of analgesic:						
	In the 1^st^ p.o.	20 (66.7%)	21 (70%)	70 (49.6%)	1.0000^a^	0.9268	0.5458–1.574
					0.1086^b^	1.800	0.8962–3.615
					0.0463^c^ *	2.051	0.9975–4.218
	After 1^st^ p.o.	8 (26.7%)	6 (20%)	20 (14.2%)	0.7611^a^	1.195	0.6928–2.061
					0.1062^b^	1.857	0.9216–3.742
					0.4095^c^	1.394	0.6319–3.076
	At dressing removal	3 (10%)	0	0	0.2373^a^	2.111	1.606–2.776
					0.0050^b^ *	6.222	4.404–8.791
	Healing (time)						
	8^th^-10^th^	9 (30%)	22 (73.3%)	84 (59.6%)	0.0017^a^ *	0.4009	0.2212–0.7266
	11^th^-14^th^	21 (70%)	8	33 (23.4%)	<0.0001b*	0.2488	0.1229–0.5038
			(26.7%)		1.0000^c^	1.064	0.5153–2.195

**Notes: p.o** = postoperative; **BC** = Bacterial Cellulose; **RR** = Relative Risk; **IC** = confidence interval. Values are total number or percentage (%).

* Statistical significance if p<0.05, to a: Polyurethane≠BC phase I; **b:** Polyurethane≠BC phase II; **c:** BC phase I≠BC phase II, by the Fisher's exact test.

Most of the patients suffered discomfort (62.4%); some of them (37.6%) said that the BC dressing presence even reduced the pain. Analgesic was prescribed to approximately 50% of the patients on the first day after surgery and this dropped to 30% on the following days. Besides that, there was no analgesic indication for use during the BC dressing removal.

Clinical complications were not observed (such as ischemia, impeded voiding, penile chordee and infection). The professionals who accompanied the BC dressing and the wound healing process classified the odor and exudate as sui generis.

## DISCUSSION

Numerous variations in the type and style of dressings have been proposed (10–12). There is no consensus on the correct dressing material to be used during the post-operatory period of male patients with genital anomalies. However, an ideal wound dressing must be easily and quickly applied, it must effectively absorb the leakages of the wound, pressurize the flaps and grafts effectively, without damaging blood circulation, thus preventing hematoma formation and helping wound healing, it must protect against infections, and must be easily and painlessly removable (1, 12).

In the present study, there was no need for dressing exchange and it was possible to wash as many times as needed, including the daily bath. The BC dressing is multiperforated and transparent making it possible for caretakers and doctors to discern the existence of possible hematomas or necrosis areas which are very troublesome in male genitalia surgeries. The BC dressing adheres naturally to the wound due to its hygroscopic characteristics, providing protection to the injured area. During the wound reepithelialization the BC dressing usually starts to come off after a couple of days and falls off spontaneously when complete healing is achieved. This dressing is effective in odor control, in exudation capacity, in humidity and temperature maintenance, all required for surgical wound healing. These characteristics were similar to those in the Phase I study (9).

BC is a biomaterial, obtained by bacterial synthesis from sugarcane molasses (13), described in previous studies as a safe dressing with low toxicity (14), biocompatible (15), promoting growth and cell differentiation (17) and, thus, contributing to the healing process (9, 16, 17) and epithelialization (18). Preclinical and clinical studies have shown that this biomaterial is effective as a mechanical barrier and adjuvant in the treatment of surgical wounds (9) and in ulcerative injuries (17).

The BC dressing is easy to use (applied/removed) and during the present study no difficulty was reported in carrying out routine hygiene procedures. Caretakers, patients and doctors also described the BC as easy to remove after saline solution (0.9%) or water irrigation. On the other hand, in a previous study that had a control group using polyurethane as a dressing, three patients (10%) reported difficulty during dressing removal due to its strong adhesion, the process as being stressful and their need for analgesics (9).

In comparison, the polyurethane dressing is a plausible alternative, whereas the BC dressing can be named as intelligent (9). The dressing referred to as “intelligent” must be able to alter the wound microenvironment, inducing endogenous signaling and leading to a wound repair (19) such as cytokines and growth factors (20).

A slight hyperemia limited to the surgical scar area was observed during the first three days with no other local irritation signs. The pain and discomfort reported by most patients can be regarded as an individual response and is possibly related to the surgical trauma. However, some patients reported pain relief using the BC dressing (37.6%). There were no reports of any adverse events. Similar BC dressing outcomes data were observed in phase I (9).

The potential disadvantages (such as ischemia, infection and pain during dressing removal) identified in another study (12) were not observed when the BC dressing was used.

This dressing provided effective pressure in hypospadias or epispadias surgery; it was effective in preventing mucosal bleeding after circumcision and provided a perfect healing in Peyronie's disease patients. In most patients, the BC dressing spontaneously fell off (71%) and the healing process was accomplished between 8^th^ and 10^th^ postoperative day (60%).

Although there is still controversy about the need for a post-operative dressing, mainly regarding hypospadias repair (13, 21), in the present study the BC proved to be a satisfactory wound dressing because it removes exudate material, creates a moist environment, offers protection from foreign substances and promotes tissue regeneration (Table-2).

The estimated cost of the BC dressing (US$4 per patient) is low when compared to similar materials (ranging from US$17 to US$25) or others, such as polyurethane (ranging from US$5 to US$7).

## CONCLUSIONS

The absence of adverse events confirms BC dressing safety and its efficacy can be validated by the suitable healing time, adhesiveness in the wound area, retention of the moisture, transudation capacity and pain and discomfort relief. The biomaterial is a highly satisfactory alternative, since it is a natural product obtained from renewable source, low cost and its use is adequate for postoperative wound healing in the urogenital area.
